# Pathological Chemotherapy Response Score in Patients Affected by High Grade Serous Ovarian Carcinoma: The Prognostic Role of Omental and Ovarian Residual Disease

**DOI:** 10.3389/fonc.2019.00778

**Published:** 2019-08-19

**Authors:** Angela Santoro, Giuseppe Angelico, Alessia Piermattei, Frediano Inzani, Michele Valente, Damiano Arciuolo, Saveria Spadola, Antonino Mulè, Piercarlo Zorzato, Anna Fagotti, Giovanni Scambia, Gian Franco Zannoni

**Affiliations:** ^1^Unità di Gineco-patologia e Patologia Mammaria, Dipartimento Scienze della Salute della Donna, del Bambino e di Sanità Pubblica, Fondazione Policlinico Universitario A. Gemelli IRCCS, Rome, Italy; ^2^Unità di Ginecologia Oncologica, Dipartimento Scienze della Salute della Donna, del Bambino e di Sanità Pubblica, Fondazione Policlinico Universitario A. Gemelli IRCCS, Rome, Italy; ^3^Istituto di Clinica Ostetrica e Ginecologica, Università Cattolica del Sacro Cuore, Rome, Italy; ^4^Istituto di Anatomia Patologica, Università Cattolica del Sacro Cuore, Rome, Italy

**Keywords:** chemotherapy response score, high-grade serous carcinoma, ovarian and omental surgical specimens, platinum-based chemotherapy, ovarian cancer

## Abstract

**Background:** The chemotherapy response score (CRS) has emerged as a simple and reproducible histopathological grading system for assessing chemotherapy response in patients affected by ovarian high-grade serous carcinoma.

**Objective:** To evaluate the prognostic impact of histological tumor response in ovarian and omental surgical specimens from patients with advanced stage ovarian high-grade serous carcinoma.

**Study Design:** A cohort of 161 women were identified from the database of Department of Gynecology, “Fondazione Policlinico Universitario Agostino Gemelli IRCCS” of Rome, Italy between January 2014 and December 2017 with a follow-up of 65 months. All the omentum, the ovarian tissue and peritoneal samples, defined as “other sites,” were reviewed by gynecological pathologists to assign a CRS of 1–3 to the omentum and ovarian sites and a score of 0–1 to the peritoneal tissue. The Cox proportional hazards regression and the log-rank test were used to assess the survival pattern and the prognostic value of the CRS adjusting for age and stage. The Kaplan-Meier method was applied to estimate the progression free and overall survival.

**Results:** The evaluation of adnexal disease showed significant differences in PFS, both in univariate and in multivariate analyses. On PFS univariate analysis, ovCRS1 vs. ovCRS3: HR, 2.27; 95% CI, 1.37–3.77; *p* = 0.001; ovCRS2 vs. ovCRS3: HR, 1.83; 95% CI, 1.03–3.23; *p* = 0.04, and on PFS multivariate model ovCRS1 vs. ovCRS3; HR, 2.53; 95% CI, 1.5–4.24; *p* = 0.001 and ovCRS2 vs. ovCRS3; HR, 1.90; 95% CI, 1.08–3.37; *p* = 0.03. Regarding the omental residual disease, as expected, CRS showed a significant prognostic value for OS and PFS; in detail the median PFS of patients with CRS1, 2 and 3 was 15, 15, and 22 months, respectively, the median OS was 41 and >50 months, respectively. Moreover, the univariate analysis for OS suggested that in our cohort the “other sites” score of 0 was significantly associated with an improvement in overall survival compared to score 1.

**Conclusions:** We demonstrated for the first time the prognostic significance of adnexal CRS confirming also the prognostic role of omental CRS.

## Introduction

Epithelial ovarian cancer (EOC) is a lethal gynecologic malignancy, and its incidence and mortality are constantly increasing. The majority of women affected by ovarian cancer present with advanced disease and their 5-years survival rate is <30% (1).

Currently, the main treatment options for OC are represented by primary debulking surgery and neoadjuvant chemotherapy (NACT) followed by surgery (interval debulking surgery, IDS). The latter has been increasingly used given the favorable results observed in two randomized controlled phase III trials which demonstrated similar progression-free survival (PFS) and overall survival (OS) after neoadjuvant chemotherapy and interval debulking surgery compared with surgery alone (2, 3).

To date, there is no uniform consensus regarding the histopathological grading system for assessing NACT response in EOC. Several studies have proposed regression grading systems demonstrating a correlation with survival. However, their findings have shown little reproducibility and have not been validated in an independent external cohort (4–9).

Recently, Böhm et al. reported a simple and reproducible histopathological grading system for assessing NACT response in EOC called CRS (chemotherapy response score) (10). CRS was tested in two independent cohorts of EOC patients treated with IDS. It consisted of a three-tier chemotherapy response score (CRS) based on omental assessment of residual disease which predicted progression-free survival and overall survival.

Albeit further studies are needed to validate these findings, the International Collaboration on Cancer Reporting has recommended the use of the CRS system for the grading of NACT response in OC (11).

Despite the significant correlation between outcome and omental disease reported by Böhm et al., when CRS was tested on adnexal residual disease, the authors did not find a statistically significant association. Moreover, similar results were obtained from another recent study.

The aim of the present study was to validate the prognostic role of the CRS system in an independent cohort of patients with advanced EOC treated with NACT followed by IDS. Our main goal was to determine the impact of pathological response to NACT especially in the primary site of tumor (ovarian residual disease) where other studies have failed to demonstrate a statistically significant correlation with outcome.

## Materials and Methods

### Patient Selection and Clinical Data

A cohort of 161 patients affected by advanced-stage (FIGO III-IV) high grade serous ovarian carcinoma between January 2014 and December 2017 was identified from the databases of our institution (Department of Gynecology, “Fondazione Policlinico Universitario Agostino Gemelli IRCCS,” Rome, Italy). Since the use of the CRS has been validated and recommended only for high grade serous carcinoma, all other EOC histotypes were initially excluded from the present study (11).

The study complied with the Ethical Principles for Medical Research Involving Human Subjects according to the World Medical Association Declaration of Helsinki and was approved by the Committee of the Applicable Institution of the “Fondazione Policlinico Universitario Agostino Gemelli IRCCS,” Rome.

All patients received neoadjuvant chemotherapy as a combination of carboplatin plus paclitaxel, followed by IDS.

According to the surgical scoring system for the IDS residual disease (0, no residual disease; 1, ≤ 1 cm residual disease; 2, >1 cm residual disease; 3, Unknown), all patients were considered score 0.

All patients were routinely evaluated with clinical visits and CT-scan examination after three cycles of NACT and, if there was evidence of progressive disease, the IDS was proposed after the third cycle.

In detail, routine follow-up was scheduled for all patients every 3–4 months for 2–3 years after finishing initial treatment and every 6 months for the next 3 years, then once a year after completion of adjuvant chemotherapy.

All the follow-up information was collected to minimize data loss and telephone-based reviewing was adopted for missing data.

### Pathology Evaluation

According to the three-tiered (CRS) proposed by Böhm et al. the respective CRS in the omental and ovarian sites were determined to measure the response to therapy in post-treatment tissue (10). Instead, the “other sites” surgical samples included peritoneal implants, visceral and lymph-nodes metastases.

All the omentum, the ovarian and the “other sites” tissue blocks that had previously been diagnosed as stage IIIC and IV EOC during the primary debulking surgery were sectioned at 4–5 μm intervals, stained with haematoxylin and eosin (H&E) and reviewed by a team of experienced gynecopathologists to assign the CRS of 1–3 for the omentum and the adnexal samples and a score of 0–1 for the peritoneal samples defined as “other sites.”

Briefly, the CRS score is summarized as follows: Score 1: No or minimal tumor response (mainly viable tumor with no or minimal regression-associated fibro-inflammatory changes, limited to a few foci); Score 2: Partial tumor response (multifocal or diffuse regression associated fibro-inflammatory changes, with viable tumor ranging from diffuse sheets, streaks or nodules, to extensive regression with multifocal but easily identifiable residual tumor; Score 3: Complete or near-complete response (mainly regression, with few irregularly scattered individual tumor cells or cell groups, all measuring <2 mm, or no residual tumor identified).

For the “other sites” samples we assigned a score of 0 for no residual disease and a score of 1 for the presence of residual disease.

Pathological review of all surgical specimens and the CRS evaluation were performed by three expert gynecologic pathologists (GFZ, AS, and GA) who were blind to clinical data and each other results. The evaluation of all cases was unanimous.

### Statistical Analysis

The Kaplan-Meier analysis was used to estimate the survival outcomes and draw the Progression-free survival (PFS) and Overall survival (OS) curves. PFS was defined as the time between the first NACT and the time of first observation of disease recurrence or death from any cause or, for patients who did not have clinical progression within the study census date, at the last follow-up consultation. OS was defined as the time from first NACT to death from any cause. To assess the prognostic value of the CRS the curves were compared using the log-rank test. Patients alive or lost to follow-up were censored. Univariate and multivariate Cox regression hazards models were performed using the SPPS Statistics 23 software (SPSS Inc, USA). Statistical significance was defined when *p* < 0.05. The sample size was determined in order to achieve a power of 0.80, an alpha of 0.05 and the hazard ratio of 2 between the two groups.

## Results

### Patient Baseline Characteristics

A total of 161 women (mean age 62 years, age range 42–86 years) with advanced stage III-IV tubo-ovarian high-grade serous carcinoma treated with neoadjuvant chemotherapy and interval debulking surgery were identified and included in the study. In detail, 138 patients (85.7%) had stage IIIC disease, and 23 (14.3%) had stage IV disease. In our study cohort, 79, 29, and 53 patients had omental CRS of 1, 2, and 3, respectively; 87, 40, and 34 patients had ovarian CRS of 1, 2, and 3, respectively; 51 and 110 patients had “other sites” score of 0 and 1 respectively. At the census date of August 1, 2018, 123 (76%) patients had recurred and 40 (24%) had died of disease.

### Univariate and Multivariate Analysis of PFS and OS for HGSC Patients

In order to evaluate the prognostic value of three-tier CRS system applied to omental and adnexal sites, the univariate and multivariate analysis for PFS and OS was performed.

The *univariate analysis* for PFS (Table 1) showed that patients classified with CRS1 or CRS2 (omental and/or ovarian disease) had a significantly higher cumulative risk of progression compared with CRS3 patients (omCRS1 vs. omCRS3: HR, 2; 95% CI, 1.32–3.02; *p* = 0.001; omCRS2 vs. omCRS3: HR, 2.03; 95% CI, 1.21–3.41; *p* = 0.007; ovCRS1 vs. ovCRS3: HR, 2.27; 95% CI, 1.37–3.77; *p* = 0.001; ovCRS2 vs. ovCRS3: HR, 1.83; 95% CI, 1.03–3.23; *p* = 0.04). Also, we demonstrated that women with “other sites” score 1 showed a significantly increased risk of progression then women with score 0 (“other sites” score 1 vs. “other sites” score 0: HR, 1.63; 95% CI, 1.11–2.39; *p* = 0.013) and that age and FIGO stage were not significantly associated with PFS.

**Table 1:** Univariate analysis for PFS.

	**Progression free survival**			
	**Univariate analysis**			
**Factor**	***N***	**HR**	**95% CI**	***p*-value**
**Omentum CRS**				
Score 1	79	2	1.32–3.02	**0.001**
Score 2	29	2.03	1.21–3.41	**0.007**
Score 3	53	ref	–	–
**Ovaries CRS**				
Score 1	87	2.27	1.37–3.77	**0.001**
Score 2	40	1.83	1.03-3.23	**0.04**
Score 3	34	ref	–	–
**Other sites**				
Score 0	51	ref	–	–
Score 1	110	1.63	1.11–2.39	**0.013**
Age at diagnosis	161	1	0.99–1.03	0.37
**FIGO stage**				
IIIC	138	ref	–	–
IV	23	1.45	0.89–2.38	0.14

*Ref indicates the group toward which is estimated the increase of cumulative risk of the event to happen, as indicated by Hazard risk (HR) of the other groups. For definition, the HR of the referece group is equal to 1*.

The *multivariate Cox regression for PFS* (Table 2), adjusted for age and FIGO stage demonstrated that an unfavorable outcomes was significantly higher in patients with omental and ovarian CRS1/CRS2 compared with those with CRS3 (omCRS1 vs. omCRS3: HR, 2.17; 95% CI, 1.41–3.33; *p* = 0.0004; omCRS2 vs. omCRS3: HR, 2.34; 95% CI, 1.35–4.04; *p* = 0.002; ovCRS1 vs. ovCRS3: HR, 2.53; 95% CI, 1.50–4.24; *p* = 0.001, ovCRS2 vs. ovCRS3: HR, 1.90; 95% CI, 1.08–3.37; *p* = 0.03).

**Table 2:** Multivariate analysis for PFS.

	**Progression free survival**			
	**Multivariate analysis**			
**Factor**	***N***	**HR**	**95% CI**	***p*-value**
**Omentum CRS**				
Score 1	79	2.17	1.41–3.33	**0.0004**
Score 2	29	2.34	1.35–4.04	**0.002**
Score 3	53	**ref**	–	–
Age at diagnosis	161	1	0.99–1.02	0.98
**FIGO stage**				
IIIC	138	**ref**	–	–
IV	23	1.88	1.12–3.14	**0.016**
**Ovaries CRS**				
Score 1	87	2.53	1.5–4.24	**0.001**
Score 2	40	1.90	1.08–3.37	**0.03**
Score 3	34	**ref**	–	–
Age at diagnosis	161	1	0.99–1.02	0.34
**FIGO stage**				
IIIC	138	**ref**	–	–
IV	23	1.77	1.06–2.93	**0.03**
**Other sites**				
Score 0	51	ref	–	–
Score 1	110	1.78	1.2–2.64	**0.004**
Age at diagnosis	161	1	0.99–1.02	0.21
**FIGO stage**				
IIIC	138	**ref**	–	–
IV	23	1.63	0.99–2.69	0.055

*Ref indicates the group toward which is estimated the increase of cumulative risk of the event to happen, as indicated by Hazard risk (HR) of the other groups. For definition, the HR of the reference group is equal to 1*.

Furthermore, although age isn't an independent prognostic factor associated with PFS, regarding FIGO stage we observed that the FIGO IV was significantly associated with a worst progression-free survival (FIGO IV vs. FIGO IIIC: HR, 1.88; 95% CI, 1.12–3.14; *p* = 0.016, when omCRS was assessed; and FIGO IV vs. FIGO IIIC: HR, 1.77; 95% CI, 1.06–2.93; *p* = 0.03, when ovCRS was assessed).

The *univariate analysis for OS* (Table 3) suggested that in our cohort the omental CRS3 as well as the “other sites” score 0 were significantly associated with an improvement in OS compared to CRS1/2 and score 1, respectively.

**Table 3:** Univariate analysis for OS.

	**Overall survival**			
	**Univariate analysis**			
**Factor**	***N***	**HR**	**95% CI**	***p*-value**
**Omentum CRS**				
Score 1	79	2.61	1.25–5.47	**0.01**
Score 2	29	1.30	0.44–3.80	0.64
Score 3	53	**ref**	–	–
**Ovaries CRS**				
Score 1	87	1.61	0.73–3.56	0.24
Score 2	40	0.64	0.21–1.95	0.43
Score 3	34	**ref**	–	–
**Other sites**				
Score 0	51	**ref**	–	–
Score 1	110	2.33	1.13–4.79	**0.02**
Age at diagnosis	161	1	0.97–1.03	0.84
**FIGO stage**				
IIIC	138	**ref**	–	–
IV	23	1.44	0.64–3.26	0.38

*Ref indicates the group toward which is estimated the increase of cumulative risk of the event to happen, as indicated by Hazard risk (HR) of the other groups. For definition, the HR of the reference group is equal to 1*.

Similarly to Böhm et al. our *multivariate analysis for OS* (Table 4) showed that the CRS system was not significant except for the omental disease. As a matter of fact patients with omCRS1 showed a significantly worst overall risk compared with omCRS3 (omCRS1 vs. omCRS3: HR, 2.75; 95% CI, 1.29–5.86; *p* = 0.01).

**Table 4:** Multivariate analysis for OS.

	**Overall survival**			
	**Multivariate analysis**			
**Factor**	***N***	**HR**	**95% CI**	***p*-value**
**Omentum CRS**				
Score 1	79	2.75	1.29–5.86	**0.01**
Score 2	29	1.45	0.47–4.44	0.5
Score 3	53	**ref**	–	–
Age at diagnosis	161	1	0.96–1.02	0.7
**FIGO stage**				
IIIC	138	**ref**	–	–
IV	23	1.54	0.68–3.57	0.31
**Ovaries CRS**				
Score 1	87	1.65	0.75–3.65	0.21
Score 2	40	0.65	0.21–1.99	0.45
Score 3	34	**ref**	–	–
Age at diagnosis	161	1	0.97–1.03	0.95
**FIGO stage**				
IIIC	138	**ref**	–	–
IV	23	1.49	0.65–3.41	0.34
**Other sites**				
Score 0	51	1	–	–
Score 1	110	2.52	1.21–5.26	**0.013**
Age at diagnosis	161	1	0.97–1.03	0.78
**FIGO stage**				
IIIC	138	**ref**	–	–
IV	23	1.73	0.75–4.03	0.20

*CI, confidence interval; CRS, chemotherapy response score; FIGO stage, International federation of Gynecology Obstetrics; HR, hazard ratio. Ref indicates the group toward which is estimated the increase of cumulative risk of the event to happen, as indicated by Hazard risk (HR) of the other groups. For definition, the HR of the reference group is equal to 1*.

Interestingly, in multivariate analysis our study highlights the significant value of the “other sites” score in both PFS and OS (Tables 2, 4).

**Figure 1:**
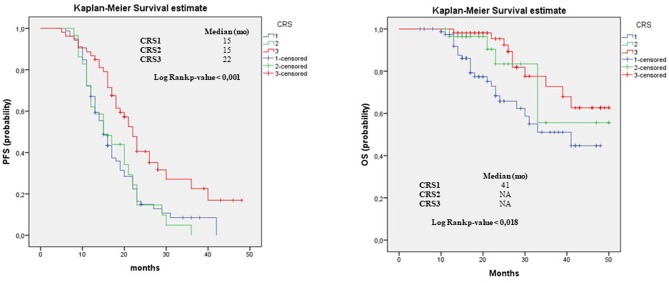
Progression free survival and overall survival in omentum. PFS and OS outcomes stratified according to the to three-tier scoring evaluation of omentum for patients who received NACT. Results of median and the log-rank test are shown.

**Figure 2:**
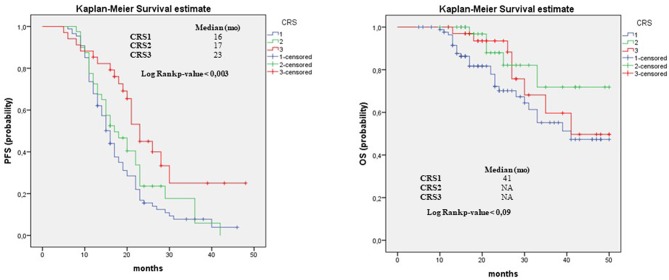
Progression Free Survival and Overall Survival in Ovarian Tissue. PFS and OS outcomes stratified according to the to three-tier scoring evaluation of ovarian for patients who received NACT. Results of median and the log-rank test are shown.

**Figure 3:**
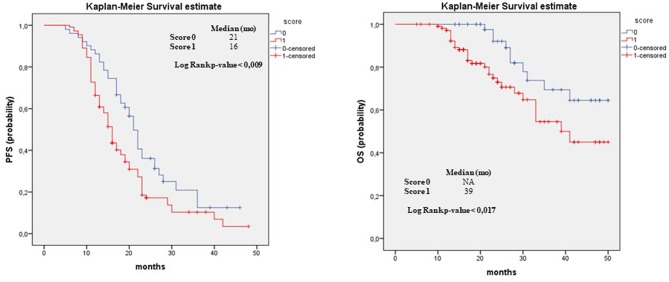
Progression free survival and overall survival in “Other Tissue.” PFS and OS outcomes stratified according to the to two-tier scoring evaluation of “other tissue” for patients who received NACT. Results of median and the log-rank test are shown.

The *Kaplan-Meier survival curves*, documenting PFS and OS outcomes stratified according to the CRS evaluation of omentum, ovary and the pathological status of “other sites,” are showed in Figures 1–3 respectively. In detail, when examining omental samples, patients with a CRS1 had a significantly shorter PFS and OS compared with those with a CRS3 (median PFS, 15 vs. 22 months; median OS, 41 vs. >50 months). Also, in ovarian samples patients with a CRS1 had a significantly shorter PFS and OS compared with those with a CRS3 (median PFS, 16 vs. 23 months; median OS, 41 vs. >50 months).

When examining “other sites” score, the Kaplan-Meier confirmed the poor prognosis in term of PFS and OS for score 1 patients compared to score 0 (median PFS, 16 vs. 21 months; median OS, 39 vs. >50 months).

## Discussion

Histopathologic response to NACT is emerging as a powerful prognostic indicator in patients affected by EOC, but to date, there is still no uniform consensus regarding the histopathological grading system for assessing NACT response in EOC. Several studies have attempted to establish pathologic regression grading systems after NACT in EOC however, their findings have shown little reproducibility and the prognostic role of pathologic response in EOC is still not consolidated (4–9).

In 2015, Böhm et al. developed a CRS three-tier grading system based on omental assessment of residual disease after NACT in a cohort of 71 EOC patients (10).

This scoring system was recently included into the International Collaboration on Cancer Reporting (ICCR) and the College of American Pathologists (CAP) guidelines for histopathologic reporting of ovarian carcinoma (11). Moreover, several studies have validated the CRS system in external cohorts of EOC patients (12–17). Interestingly, in the last study on this topic, published recently by Rajkumar (17), there was no significant distinction in both progression free survival and overall survival estimates between women with CRS1 and CRS2, therefore the Authors proposed a binary prognostication system (CRS 3 vs. CRS 1/2) as opposed to a three-point score.

However, as initially observed by Böhm et al. (10), adnexal residual disease after chemotherapy has been reported as difficult and less reproducible to score and has shown no correlation with outcome.

In the present paper we successfully validated the CRS system in an independent cohort of 161 EOC patients demonstrating for the first time the prognostic significance of adnexal CRS.

In detail, when evaluating adnexal disease, significant differences in PFS were observed between CRS1, CRS2, and CRS3 groups, both in univariate and in multivariate analyses (univariate model—ovCRS1 vs. ovCRS3: HR, 2.27; 95% CI, 1.37–3.77; *p* = 0.001; ovCRS2 vs. ovCRS3: HR, 1.83; 95% CI, 1.03–3.23; *p* = 0.04; multivariate model—ovCRS1 vs. ovCRS3; HR, 2.53; 95% CI, 1.5–4.24; *p* = 0.001 and ovCRS2 vs. ovCRS3; HR, 1.90; 95% CI, 1.08–3.37; *p* = 0.03).

Regarding omental residual disease, as expected, CRS showed a significant prognostic value for OS and PFS; in detail the median PFS of patients with CRS1, 2 and 3 was 15, 15, and 22 months, respectively, the median OS was 41 and >50 months, respectively. Moreover, as Böhm et al. (10) and Rajkumar (17), we observed no significant differences in terms of OS and PFS between omental CRS1 and omental CRS2 tumors. Conversely, we have found significant differences between ovCRS1 and ovCRS2 in terms of OS being the median OS for ovCRS1 patients 41 months vs. a median OS of >50 months observed for ovCRS2 patients (*p* = 0.04). However, no significant differences were observed in terms of PFS between ovCRS1 and ovCRS2 groups.

Nonetheless, based on our findings, we suggest maintaining the three-point score prognostication system.

Moreover, when we evaluated the combination of omental and adnexal CRS for each patient, we observed a moderate but not statistically significant trend for longer PFS for patients with omental CRS1 and ovarian CRS2 compared to patients with omental CRS2 and ovarian CRS1. In detail patients with omental CRS1 and adnexal CRS2 showed a mean PFS of 15 months vs. a mean PFS of 10 months observed for patients with omental CRS2 and ovarian CRS1. This trend indicates that an absent or minimal tumor response in the adnexal samples have higher potential to influence the prognosis when compared to an absent response in omental samples.

However, when we compared CRS2 and CRS3 scores in omental and ovarian samples we observed a trend for better PFS in patients with omental CRS3 and ovarian CRS2 (mean PFS 36 months) compared to patients with omental CRS2 and ovarian CRS3 (mean PFS 21 months). Therefore, a partial tumor response in omental samples seems to be related with a worse outcome when compared to a partial response in adnexal samples.

Finally, as expected, we observed significant differences in terms of survival also for the other sites samples. In fact patients with a score of 0 showed a better outcome (median PFS 21 months, median OS >50 months) compared to patients with a score of 1 (median PFS 16 months, median OS 39 months).

Another interesting result was represented by the improved survival for FIGO Stage IIIC patients compared to FIGO Stage IV in statistical analysis. From these data, FIGO IV patients showed a worse progression-free survival compared to FIGO IIIC patients (Table 2).

In this way, our results are in line with a recent meta-analysis in which authors observed a slight increase in OS and PFS for Stage III patients compared to stage IV (although without statistical significance) (18). However, conflicting results still exist in literature since EORTC 55971 trial suggested that Stage IIIC patients showed more benefits from primary debulking surgery, whereas stage IV patients showed more benefits from NACT-IDS (2). Further works should be encouraged to identify chemotherapeutic factors that increase the chance of complete pathological response in resected patients in advanced tumoral stage.

Finally, the strengths of our work are represented by external validation of the CRS system in an independent large cohort of advanced stage, ovarian HGSCs. In this study the CRS system emerged as a useful prognostic tool to stratify EOC patients. Moreover, the present study is the first to validate the CRS also for ovarian residual disease where other authors have failed to demonstrate a statistically significant correlation with outcome. Anyway, the study presented several limitations, including its retrospective design and the monocentric nature. Furthermore, we were not able to collect information about BRCA1/2 mutations which are well-known predictive markers of platinum response. Another refinement could be the separation of CRS 3 category in two groups: complete pathological response vs. microscopic residual disease. Although we did not perform this possible prognostic separation, future works may demonstrate its clinical value, incorporating also this sub-classification into routine practice.

In conclusion, our study confirms that CRS represents a possible surrogate to early predict patient survival and risk of early relapses and a clinical opportunity to personalize treatment. In particular, some Authors suggest that CRS 3 women will not relapse at 5 years, representing optimal candidates for additional adjuvant therapeutic agent such as poly (adenosine diphosphate–ribose) polymerase (PARP) inhibitors (19). Conversely, CRS1/2 tumors, that frequently recurring within 5 years, should be selected and incorporated immediately into trials of new therapeutical regimen. We would encourage the development of a model to predict prognosis, incorporating CRS but also radiological and biochemical response, surgical residual disease, tumor immune profile and microenvironment, and molecular classification.

## Ethics Statement

This study complied with the Ethical Principles for Medical Research Involving Human Subjects according to the World Medical Association Declaration of Helsinki and was approved by the Committee of the Applicable Institution of the Fondazione Policlinico Universitario Agostino Gemelli IRCCS, Rome.

## Author Contributions

GZ conceived and designed the experiments. AS, GA, AM, and FI contributed equally to the design of the work. FI, MV, DA, SS, and PZ contributed to data collection. AP performed the data analysis. GZ, AS, AP, AF, and GS wrote the paper. GZ and GS revised the entire work.

### Conflict of Interest Statement

The authors declare that the research was conducted in the absence of any commercial or financial relationships that could be construed as a potential conflict of interest.
